# Crystal water as the mol­ecular glue for obtaining different co-crystal ratios: the case of gallic acid tris-caffeine hexa­hydrate

**DOI:** 10.1107/S2056989018004528

**Published:** 2018-03-27

**Authors:** L. Vella-Zarb, U. Baisch

**Affiliations:** a University of Malta, Msida, MSD 2080, Malta

**Keywords:** crystal structure, organic co-crystal, hydrate, gallic acid, caffeine

## Abstract

The crystal structure of the hexa­hydrate co-crystal of gallic acid and caffeine, C_7_H_6_O_5_·3C_8_H_10_N_4_O_2_·6H_2_O or **GAL3CAF·6H_2_O**, is a remarkable example of the importance of hydrate water acting as structural glue to facilitate the crystallization of two components of different stoichiometries and thus to compensate an imbalance of hydrogen-bond donors and acceptors. The water mol­ecules provide the additional hydrogen bonds required to form a crystalline solid. Whereas the majority of hydrogen bonds forming the inter­molecular network between gallic acid and caffeine are formed by crystal water, only one direct classical hydrogen bond between two mol­ecules is formed between the carb­oxy­lic oxygen of gallic acid and the carbonyl oxygen of caffeine with *d*(*D*⋯*A*) = 2.672 (2) Å. All other hydrogen bonds either involve crystal water or utilize protonated carbon atoms as donors.

## Abstract 2

This co-crystal structure consists of three caffeine mol­ecules and one gallic acid mol­ecule as well as six hydrate water mol­ecules per formula unit. It can be described as being composed of two types of mol­ecular layers connected *via* hydrogen-bonding inter­actions to solvent water. The two layers stack in an alternate manner between layers consisting solely of caffeine mol­ecules and layers of caffeine and gallic acid mol­ecules.

## Chemical context   

Gallic acid and its derivatives are widely known compounds in the pharmaceutical and chemical industry (Nayeem *et al.*, 2016 ▸; Clarke *et al.*, 2011 ▸). One such example is the dietary polyphenol found in *Choerospondiatis fructus*, a Mongolian medicinal herb used to treat conditions such as angina pectoris (Zhao *et al.*, 2007 ▸). Lately, it has gained a lot of attention as a versatile component in crystal enineering, in particular with regards to co-crystallization and hydratation. Gallic acid could represent an entire microcosm of the special challenges and opportunities afforded by hydrates (Clarke *et al.*, 2011 ▸) as it contains two of the most ubiquitous functional groups present in APIs: carb­oxy­lic acids and phenols. As part of a series of co-crystallization experiments in which both caffeine and gallic acid were used as coformers, single crystals of hydrated gallic acid and caffeine **GAL3CAF·6H_2_O** in the ratio gallic acid:caffeine:water of 1:3:6 were obtained and characterized by single-crystal X-ray diffraction. The crystal structure is reported herein and compared to the different hydrated forms of this co-crystal **GALCAF·0.5H_2_O** reported elsewhere (Clarke *et al.*, 2010 ▸). The crystal structures differ greatly because of the different stoichiometry of the coformers. The different number of water mol­ecules is necessary to act as structural glue, thereby facilitating crystallization.

## Structural commentary   

**Figure 1:**
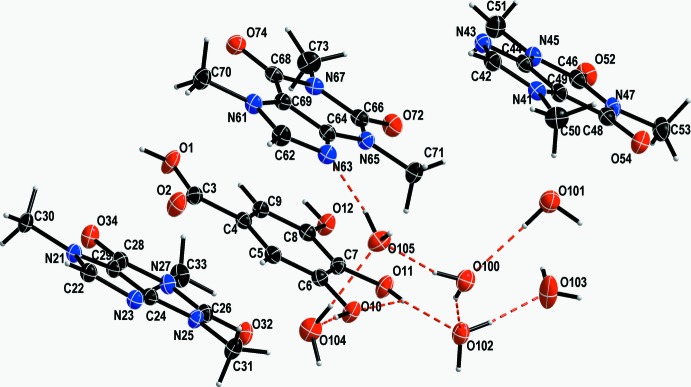
Mol­ecular structure of the **GAL3CAF·6H_2_O** showing the labelling scheme and displacement ellipsoids drawn at 50% probability level.

The asymmetric unit of the co-crystal **GAL3CAF·6H_2_O** consists of three independent caffeine mol­ecules and one gallic acid mol­ecule as well as six hydrate water mol­ecules. Gallic acid can be described as a thrice-substituted benzoic acid with hydroxyl groups in both the *meta* and *para* positons. Caffeine consists of a purine backbone with carbonyl substit­uents at positions 2 and 6 (C26, C28, C46, C48, C66, C68) and methyl groups connected to three out of four nitro­gen atoms (Fig. 1 ▸).
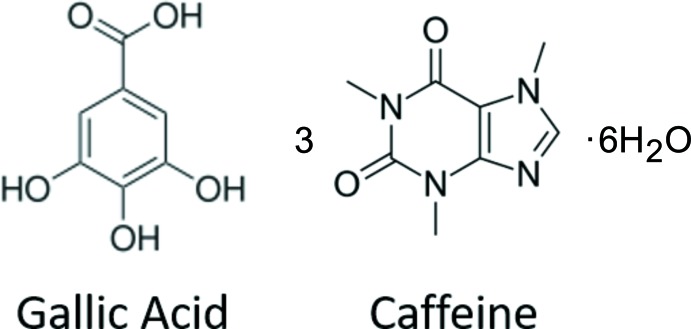


Bond distances of the aromatic rings and substituents of both types of mol­ecules lie within the expected ranges and exhibit the usual lengths for aromatic, double or single homo- or heteroatomic bonds. Only one nitro­gen atom on each of the three caffeine mol­ecules can act as a hydrogen-bond acceptor (N23, N43, N63) with the protonated carbon in the five-membered ring (C22, C42, C62) acting as a weak hydrogen-bond donor. Every mol­ecule exhibits weak intra­molecular inter­actions (Steiner, 2002 ▸). Whereas gallic acid forms inter­moleclar bonds between two adjacent hydroxyl substituents [O11—H11⋯O10, *d*(*D*⋯*A*) = 2.709 (2) Å], the caffeine mol­ecules form weak inter­actions between two of three methyl carbons and two carbonyl oxygen or backbone nitro­gen atoms, namely C31, C33, C51, C53, C71, C73 as well as O32, N43, O54, O72, and O74. Distances range between 2.71 and 2.95 Å. In comparison, the corresponding intra­molecular hydrogen-bonding inter­actions in the published **GALCAF·0.5H_2_O** structure reported in the Cambridge Structural Database (Groom *et al.*, 2016 ▸) with code MUPNOB (Clarke *et al.*, 2010 ▸) have distances of *d*(*D*⋯A)_gallic acid_ = 2.743 (2), 2.712 (2) Å and *d*(*D*⋯*A*)_caffeine_ of 2.78–2.71 Å.

## Supra­molecular features   

As a result of the limited number of hydrogen-bond donors and acceptors in both gallic acid and caffeine, the packing strongly depends on (i) the concentration of each of the components in solution as well as (ii) other experimental conditions such as other components in solution, temperature, pressure, *etc*. In fact, there is a large difference in the way both mol­ecules pack in the crystal lattice.

The crystal structure of **GALCAF·0.5H_2_O** (Clarke *et al.*, 2010 ▸) has a 1:1:0.5 ratio of gallic acid, caffeine and water mol­ecules. Both mol­ecules form hydrogen-bonded tapes that are built by COO—H⋯N and O—H⋯O inter­actions [O⋯N = 2.705 (2) Å and O⋯O = 2.703 (2) and 2.750 (2) Å] formed between the hydoxyl substituents on gallic acid mol­ecules and the carbonyl moieties of adjacent caffeine mol­ecules. These tapes are then cross-linked by water mol­ecules that hydrogen-bond with the third hy­droxyl group in each gallic acid mol­ecule [O⋯O = 2.857 (1) Å]. The water mol­ecules facilitate the formation of bilayers that stack in an *ABAB* manner sustained by π–π inter­actions. The distances between these layers of mol­ecules can be calculated from the distances between the centroids of the aromatic rings of the two mol­ecules and range from 3.3742 (14) to 4.3402 (14) Å. The ratio between classical hydrogen-bond donors and acceptors is 4:4 (four donors and one acceptor on the gallic acid mol­ecule and three acceptors on the caffeine mol­ecule).

**Figure 2:**
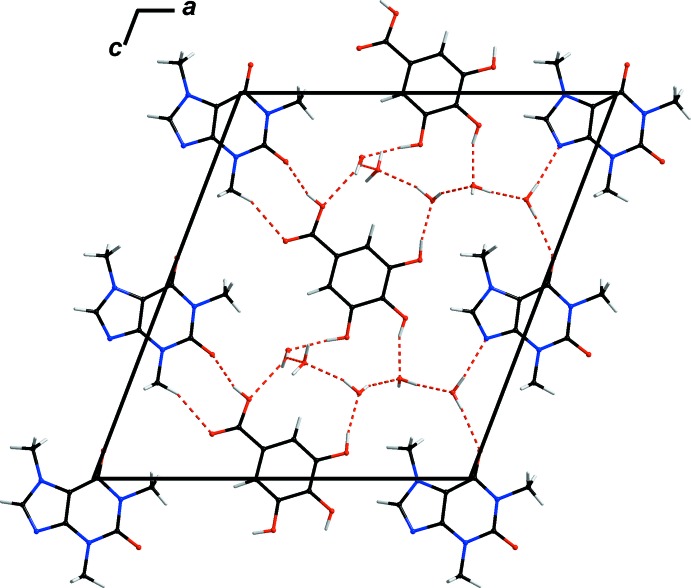
Crystal packing of **GAL3CAF·6H_2_O** viewed along *b*. Hydrogen-bonding inter­actions (Table 1 ▸) are shown as red dashed lines.

**Figure 3:**
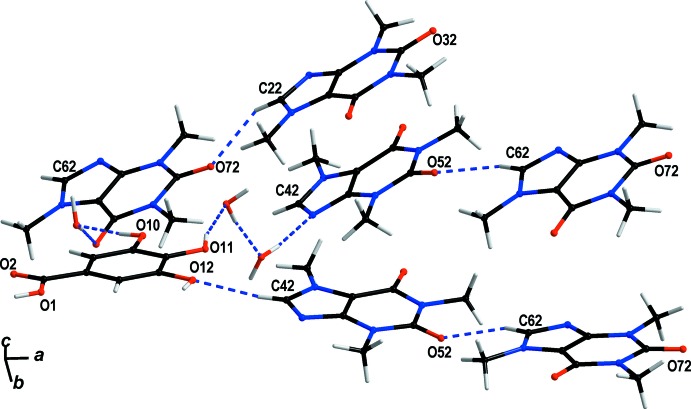
Crystal packing of **GAL3CAF·6H_2_O**. Hydrogen-bonding inter­actions (Table 1 ▸) are shown as blue dashed lines.

The different balance of gallic acid and caffeine in **GAL3CAF·6H_2_O** affects the donor/acceptor ratio significantly. There are still only four classical hydrogen-bond donors deriving from the hydroxyl groups on gallic acid, but ten hydrogen-bond acceptors (three on each caffeine and one on gallic acid). This discrepency is equilibrated by inclusion of additional solvent water mol­ecules into the crystal structure. These act as structural glue enabling crystallization in different stoichiometries and thus, compensating for the above imbalance. The water mol­ecules provide the additional hydrogen bonds required to form a crystalline solid. Thus, the majority of hydrogen bonds forming the inter­molecular network between gallic acid and caffeine are formed by crystal water (Table 1 ▸) with *d*(*D*⋯*A*) ranging from 2.643 (2) to 3.011 (2) Å. Direct classical hydrogen bonding between non-carbon atoms can only be observed between the carb­oxy­lic oxygen of gallic acid (O1) and the carbonyl oxygen of caffeine (O32) with *d*(*D⋯A*) = 2.672 (2) Å (Fig. 2 ▸). Additionally, there is a significant number of weak C—H⋯O inter­actions present between gallic acid mol­ecules and caffeine mol­ecules and between caffeine mol­ecules themselves (Table 1 ▸). One of these inter­actions is between carb­oxy­lic acid and a carbonyl oxygen *via* hydrogen bonding that is almost parallel to an inter­action between the carbonyl oxygen (O2) of the carb­oxy­lic group in gallic acid with the adjacent proton of a caffeine methyl substituent (C31) *d*(*D⋯A*) 3.326 (3) Å. Another C—H⋯O inter­action is notable as it forms a linear chain connecting all caffeine mol­ecules to each other (Fig. 3 ▸). These are formed between the only protonated carbon atom in the purine backbone (C22—H22, C42—H42, C62—H62) and the carbonyl oxygen of the next caffeine mol­ecule (O32, O52, O72) with donor–acceptor distances ranging from 3.119 (3) to 3.227 (3) Å. O12 links gallic acid mol­ecules to these chains *via* additional weak C—H⋯O inter­actions to C30 [*d*(*D⋯A*) 3.247 (3) Å] and C42 [*d*(*D⋯A*) = 3.254 (3) Å]. A comparable inter­action between the protonated carbon of the purine ring does not exist in the **GALCAF·0.5H_2_O** structure.

**Figure 4:**
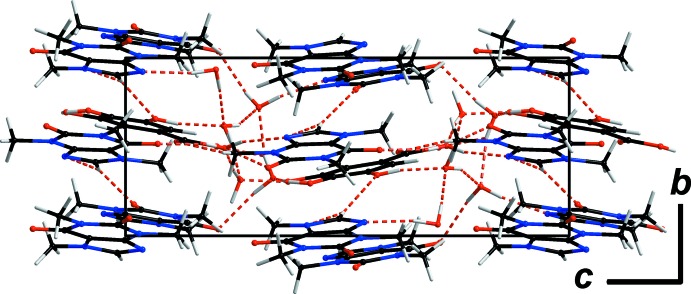
Crystal packing of **GAL3CAF·6H_2_O** viewed along *a*. Hydrogen-bonding inter­actions are shown as red dashed lines.

The crystal structure of **GAL3CAF·6H_2_O** can be described as having two types of mol­ecular layers connected *via* hydrogen-bonding inter­actions with solvent water mol­ecules. Layers consisting solely of caffeine mol­ecules are stacked alternately with layers composed of caffeine and gallic acid mol­ecules (Fig. 4 ▸). The distances between the centroids of the aromatic rings are within the significance range at 3.231 (13) and 4.5028 (13) Å. Thus π-stacking of the aromatic rings is both stronger and weaker in places.

The discussed crystal structure provides a good representation of the large impact of weak C—H⋯O inter­actions and of how solvent mol­ecules can play a crucial role in the formation of crystal structures. All our attempts at obtaining a solventless co-crystal with the same stoichiometry have failed so far.

## Synthesis and crystallization   

The crystals were obtained as a by-product in a reaction aiming for the synthesis of a lanthanide salt. Gel crystallizations were carried out in order to slow the crystallization process down. This technique involves a piece of glassware that allows two solutions to diffuse through a (tetra­methyl­orthosilicate) gel medium. The two sets of reagents then react when they eventually diffuse through the gel. Tetra­methyl­orthosilicate gel (10%) was prepared freshly from 7 mL in 63 mL distilled water using Na_2_CO_3_ to make the gel approximately pH 8, and left to set overnight using U-tubes. Solutions were put into the two reservoirs: one contained caffeine (1 mmol, 0.2 g), while a solution containing an excess gallic acid and lanthanide was in the other.

## Refinement   

Crystal data, data collection and structure refinement details are summarized in Table 2 ▸. Methyl H atoms were refined as riding (C—H = 0.98 Å with *U*_iso_(H) = 1.5*U*_eq_(C).

## Supplementary Material

Crystal structure: contains datablock(s) I. DOI: 10.1107/S2056989018004528/lh5871sup1.cif

Structure factors: contains datablock(s) I. DOI: 10.1107/S2056989018004528/lh5871Isup2.hkl

Supporting information file. DOI: 10.1107/S2056989018004528/lh5871Isup3.cml

CCDC reference: 1830592

Additional supporting information:  crystallographic information; 3D view; checkCIF report

## Figures and Tables

**Table 1:** Hydrogen-bond geometry (Å, °)

*D*—H⋯*A*	*D*—H	H⋯*A*	*D*⋯*A*	*D*—H⋯*A*
O1—H1⋯O32^i^	0.85 (3)	1.86 (3)	2.672 (2)	161 (3)
O10—H10⋯O104	0.86 (4)	1.79 (4)	2.643 (2)	174 (4)
O11—H11⋯O10	0.83 (3)	2.32 (4)	2.709 (2)	109 (3)
O11—H11⋯O102	0.83 (4)	1.88 (4)	2.680 (2)	161 (3)
O12—H12⋯O100^i^	0.86 (3)	1.86 (3)	2.702 (2)	166 (3)
O100—H10*C*⋯O105	0.83 (4)	2.07 (4)	2.851 (3)	157 (4)
O100—H10*D*⋯O10	0.82 (4)	2.74 (3)	3.286 (2)	126 (3)
O100—H10*D*⋯O102	0.82 (4)	2.02 (4)	2.798 (3)	158 (3)
O101—H10*I*⋯N43^ii^	0.94 (4)	1.97 (4)	2.898 (3)	170 (3)
O101—H10*J*⋯O100	0.88 (4)	2.09 (4)	2.960 (3)	170 (4)
O102—H10*A*⋯O101^iii^	0.89 (4)	1.87 (4)	2.754 (3)	169 (4)
O102—H10*B*⋯O103	0.85 (4)	1.85 (4)	2.691 (3)	173 (3)
O103—H10*K*⋯O34^iv^	0.78 (5)	2.06 (5)	2.832 (3)	168 (5)
O103—H10*L*⋯N23^v^	0.84 (5)	1.99 (5)	2.823 (3)	168 (4)
O104—H10*G*⋯O1^vi^	0.91 (3)	2.50 (3)	3.011 (2)	116 (2)
O104—H10*G*⋯O105	0.91 (3)	2.03 (3)	2.857 (3)	151 (3)
O104—H10*H*⋯O74^vi^	0.93 (5)	2.00 (5)	2.924 (2)	171 (4)
O105—H10*E*⋯O74^ii^	0.86 (5)	2.11 (5)	2.952 (2)	169 (4)
O105—H10*F*⋯N63	0.88 (4)	1.92 (4)	2.798 (2)	173 (4)
C22—H22⋯O72^vii^	0.97 (3)	2.38 (3)	3.227 (3)	146 (2)
C30—H30*C*⋯O12^vii^	0.98	2.71	3.247 (3)	115
C31—H31*C*⋯O2^vi^	0.98	2.40	3.326 (3)	158
C42—H42⋯O12^viii^	0.92 (3)	2.45 (3)	3.254 (3)	146 (3)
C42—H42⋯O72	0.92 (3)	2.66 (3)	3.136 (3)	113 (2)
C62—H62⋯O52^vii^	0.96 (3)	2.24 (3)	3.119 (3)	151 (3)

Symmetry codes: (i) 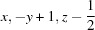; (ii) 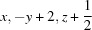; (iii) 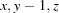; (iv) 
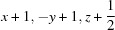
; (v) 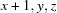; (vi) 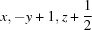; (vii) 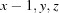; (viii) 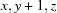.

**Table 2:** Experimental details

Crystal data	
Chemical formula	C_7_H_6_O_5_·3C_8_H_10_N_4_O_2_·6H_2_O
*M*_r_	860.81
Crystal system, space group	Monoclinic, *P* *c*
Temperature (K)	150
*a*, *b*, *c* (Å)	16.5434 (3), 6.79456 (11), 18.1390 (4)
β (°)	110.865 (2)
*V* (Å^3^)	1905.21 (7)
*Z*	2
Radiation type	Mo *K*α
μ (mm^−1^)	0.12
Crystal size (mm)	0.66 × 0.37 × 0.06

Data collection	
Diffractometer	Rigaku Oxford Diffraction Xcalibur, Atlas, Gemini ultra
Absorption correction	Analytical (*CrysAlis PRO*; Rigaku OD, 2015 ▸)
*T*_min_, *T*_max_	0.946, 0.994
No. of measured, independent and observed [*I* > 2σ(*I*)] reflections	38532, 9519, 8931
*R*_int_	0.029
(sin θ/λ)_max_ (Å^−1^)	0.695

Refinement	
*R*[*F* ^2^ > 2σ(*F* ^2^)], *wR*(*F* ^2^), *S*	0.033, 0.086, 1.04
No. of reflections	9519
No. of parameters	645
No. of restraints	2
H-atom treatment	H atoms treated by a mixture of independent and constrained refinement
Δρ_max_, Δρ_min_ (e Å^−3^)	0.24, −0.23
Absolute structure	Flack *x* determined using 4000 quotients [(*I* ^+^)−(*I* ^−^)]/[(*I* ^+^)+(*I* ^−^)] (Parsons *et al.*, 2013 ▸)
Absolute structure parameter	−0.1 (2)

Computer programs: *CrysAlis PRO* (Rigaku OD, 2015 ▸), *SHELXT* (Sheldrick, 2015*a* ▸), *SHELXL* (Sheldrick, 2015*b* ▸), *DIAMOND* (Brandenburg, 1999 ▸) and *OLEX2* (Dolomanov *et al.*, 2009 ▸).

## supplementary crystallographic information

### Crystal data

**Table:**

C_7_H_6_O_5_·3C_8_H_10_N_4_O_2_·6H_2_O	*F*(000) = 908
*M**_r_* = 860.81	*D*_x_ = 1.501 Mg m^−^^3^
Monoclinic, *P**c*	Mo *K*α radiation, λ = 0.71073 Å
*a* = 16.5434 (3) Å	Cell parameters from 27647 reflections
*b* = 6.79456 (11) Å	θ = 3.0–29.6°
*c* = 18.1390 (4) Å	µ = 0.12 mm^−^^1^
β = 110.865 (2)°	*T* = 150 K
*V* = 1905.21 (7) Å^3^	Plate, clear colourless
*Z* = 2	0.66 × 0.37 × 0.06 mm

### Data collection

**Table:**

Rigaku Oxford Diffraction Xcalibur, Atlas, Gemini ultra diffractometer	8931 reflections with *I* > 2σ(*I*)
Detector resolution: 10.3968 pixels mm^-1^	*R*_int_ = 0.029
ω scans	θ_max_ = 29.6°, θ_min_ = 3.0°
Absorption correction: analytical (CrysAlisPro; Rigaku OD, 2015)	*h* = −21→22
*T*_min_ = 0.946, *T*_max_ = 0.994	*k* = −9→9
38532 measured reflections	*l* = −25→24
9519 independent reflections	

### Refinement

**Table:**

Refinement on *F*^2^	Hydrogen site location: mixed
Least-squares matrix: full	H atoms treated by a mixture of independent and constrained refinement
*R*[*F*^2^ > 2σ(*F*^2^)] = 0.033	*w* = 1/[σ^2^(*F*_o_^2^) + (0.051*P*)^2^ + 0.2608*P*] where *P* = (*F*_o_^2^ + 2*F*_c_^2^)/3
*wR*(*F*^2^) = 0.086	(Δ/σ)_max_ < 0.001
*S* = 1.04	Δρ_max_ = 0.24 e Å^−^^3^
9519 reflections	Δρ_min_ = −0.23 e Å^−^^3^
645 parameters	Absolute structure: Flack *x* determined using 4000 quotients [(*I*^+^)-(*I*^-^)]/[(*I*^+^)+(*I*^-^)] (Parsons *et al.*, 2013)
2 restraints	Absolute structure parameter: −0.1 (2)
Primary atom site location: dual	

### Special details

**Table:**

Geometry. All esds (except the esd in the dihedral angle between two l.s. planes) are estimated using the full covariance matrix. The cell esds are taken into account individually in the estimation of esds in distances, angles and torsion angles; correlations between esds in cell parameters are only used when they are defined by crystal symmetry. An approximate (isotropic) treatment of cell esds is used for estimating esds involving l.s. planes.

### Fractional atomic coordinates and isotropic or equivalent isotropic displacement parameters (Å^2^)

**Table:**

	*x*	*y*	*z*	*U*_iso_*/*U*_eq_	
O12	0.64601 (10)	0.3426 (2)	0.43298 (9)	0.0237 (3)	
O74	0.39816 (11)	0.9622 (2)	0.29064 (9)	0.0249 (3)	
O10	0.53021 (10)	0.3054 (3)	0.63346 (9)	0.0250 (3)	
O72	0.66076 (10)	0.8227 (3)	0.48111 (10)	0.0279 (3)	
O102	0.71299 (11)	0.3149 (3)	0.74098 (9)	0.0259 (3)	
O11	0.66635 (9)	0.2955 (2)	0.58370 (9)	0.0241 (3)	
O32	0.19013 (10)	0.3831 (2)	0.67800 (9)	0.0257 (3)	
O34	−0.00090 (10)	0.4828 (2)	0.42738 (9)	0.0259 (3)	
O54	1.06258 (11)	0.9604 (3)	0.70872 (10)	0.0289 (3)	
O1	0.33315 (11)	0.4973 (3)	0.29402 (10)	0.0292 (4)	
O100	0.61941 (12)	0.6181 (3)	0.77785 (10)	0.0266 (3)	
O105	0.44257 (11)	0.7234 (3)	0.69929 (10)	0.0288 (3)	
O52	1.14078 (10)	0.9016 (3)	0.49147 (10)	0.0300 (4)	
O2	0.27018 (10)	0.4707 (3)	0.38411 (10)	0.0300 (4)	
O104	0.38756 (11)	0.3238 (3)	0.66710 (11)	0.0312 (4)	
O101	0.74791 (12)	0.9351 (3)	0.79622 (11)	0.0325 (4)	
N27	0.09392 (11)	0.4384 (3)	0.55358 (11)	0.0200 (3)	
N41	0.88046 (12)	1.0593 (3)	0.59097 (11)	0.0210 (3)	
N23	−0.09763 (11)	0.5534 (3)	0.63666 (10)	0.0203 (3)	
N67	0.52922 (11)	0.8905 (3)	0.38714 (11)	0.0201 (3)	
N21	−0.13658 (11)	0.5677 (3)	0.50514 (10)	0.0187 (3)	
N61	0.32462 (12)	0.9330 (3)	0.42452 (11)	0.0216 (4)	
N63	0.41353 (12)	0.8759 (3)	0.54870 (11)	0.0221 (4)	
O103	0.86742 (14)	0.4935 (4)	0.77642 (12)	0.0486 (6)	
N45	1.00201 (12)	0.9877 (3)	0.46922 (11)	0.0213 (4)	
N47	1.10076 (11)	0.9323 (3)	0.59887 (11)	0.0221 (4)	
N43	0.85646 (11)	1.0770 (3)	0.46163 (11)	0.0223 (4)	
N25	0.05332 (11)	0.4685 (3)	0.66583 (10)	0.0200 (3)	
N65	0.54639 (11)	0.8526 (3)	0.52143 (10)	0.0193 (3)	
C9	0.49273 (13)	0.4032 (3)	0.39853 (12)	0.0182 (4)	
C28	0.01160 (13)	0.4814 (3)	0.49805 (12)	0.0185 (4)	
C68	0.43992 (14)	0.9264 (3)	0.36060 (12)	0.0193 (4)	
C64	0.45928 (13)	0.8801 (3)	0.49996 (12)	0.0188 (4)	
C5	0.43204 (13)	0.3818 (3)	0.50144 (12)	0.0185 (4)	
C22	−0.16156 (14)	0.5854 (3)	0.56754 (12)	0.0204 (4)	
C4	0.42193 (13)	0.4139 (3)	0.42310 (12)	0.0180 (4)	
C26	0.11628 (13)	0.4278 (3)	0.63532 (12)	0.0198 (4)	
C3	0.33445 (14)	0.4627 (3)	0.36677 (12)	0.0202 (4)	
C42	0.82336 (14)	1.0955 (3)	0.51858 (13)	0.0227 (4)	
C62	0.33233 (14)	0.9082 (3)	0.49978 (13)	0.0241 (4)	
C6	0.51354 (13)	0.3398 (3)	0.55560 (12)	0.0189 (4)	
C29	−0.04957 (13)	0.5192 (3)	0.53528 (12)	0.0181 (4)	
C24	−0.02878 (13)	0.5128 (3)	0.61509 (12)	0.0182 (4)	
C8	0.57394 (13)	0.3606 (3)	0.45261 (12)	0.0184 (4)	
C69	0.40732 (13)	0.9150 (3)	0.42277 (12)	0.0185 (4)	
C7	0.58546 (13)	0.3319 (3)	0.53187 (12)	0.0183 (4)	
C49	0.95774 (13)	1.0134 (3)	0.58130 (13)	0.0190 (4)	
C48	1.04131 (14)	0.9686 (3)	0.63709 (13)	0.0217 (4)	
C46	1.08429 (14)	0.9386 (3)	0.51791 (13)	0.0219 (4)	
C30	−0.19092 (13)	0.5917 (3)	0.42252 (12)	0.0208 (4)	
H30A	−0.195314	0.465826	0.394977	0.031*	
H30B	−0.165253	0.690518	0.398138	0.031*	
H30C	−0.248743	0.634908	0.418844	0.031*	
C44	0.94005 (13)	1.0249 (3)	0.50165 (12)	0.0197 (4)	
C66	0.58361 (14)	0.8529 (3)	0.46485 (13)	0.0206 (4)	
C71	0.60075 (14)	0.8227 (3)	0.60429 (13)	0.0258 (4)	
H71A	0.578830	0.903248	0.637924	0.039*	
H71B	0.660364	0.861399	0.612352	0.039*	
H71C	0.599411	0.683589	0.618036	0.039*	
C31	0.07288 (15)	0.4634 (4)	0.75120 (13)	0.0263 (4)	
H31A	0.049780	0.341920	0.765162	0.039*	
H31B	0.046261	0.577200	0.766781	0.039*	
H31C	0.135692	0.467660	0.778760	0.039*	
C51	0.98219 (16)	0.9950 (4)	0.38378 (14)	0.0280 (5)	
H51A	1.028745	1.063988	0.372886	0.042*	
H51B	0.927512	1.065112	0.358458	0.042*	
H51C	0.977081	0.860691	0.362901	0.042*	
C50	0.86261 (17)	1.0583 (4)	0.66409 (15)	0.0284 (5)	
C33	0.16380 (14)	0.4060 (4)	0.52176 (14)	0.0259 (5)	
H33A	0.148733	0.294353	0.485177	0.039*	
H33B	0.218050	0.377880	0.565271	0.039*	
H33C	0.170850	0.524415	0.493788	0.039*	
C53	1.19054 (14)	0.8887 (4)	0.64940 (15)	0.0301 (5)	
H53A	1.225454	1.008608	0.656930	0.045*	
H53B	1.214230	0.787698	0.624216	0.045*	
H53C	1.191737	0.840579	0.700719	0.045*	
C73	0.57111 (16)	0.8896 (4)	0.32820 (15)	0.0292 (5)	
H73A	0.573261	0.754593	0.309962	0.044*	
H73B	0.630055	0.941262	0.351905	0.044*	
H73C	0.538002	0.972313	0.283348	0.044*	
H5	0.3845 (19)	0.391 (4)	0.5203 (16)	0.022 (6)*	
H9	0.4873 (17)	0.424 (4)	0.3426 (16)	0.017 (6)*	
H22	−0.220 (2)	0.629 (4)	0.5596 (17)	0.030 (7)*	
H1	0.282 (2)	0.521 (5)	0.2629 (19)	0.035 (8)*	
H12	0.631 (2)	0.369 (4)	0.3832 (19)	0.031 (7)*	
H62	0.284 (2)	0.912 (5)	0.5170 (18)	0.035 (8)*	
H10	0.482 (3)	0.308 (6)	0.641 (2)	0.061 (11)*	
C70	0.24418 (15)	0.9659 (4)	0.35795 (14)	0.0265 (4)	
H70A	0.194877	0.947555	0.375199	0.040*	
H70B	0.240186	0.871975	0.315813	0.040*	
H70C	0.243510	1.100411	0.338199	0.040*	
H42	0.767 (2)	1.131 (4)	0.5092 (18)	0.033 (7)*	
H11	0.669 (2)	0.304 (5)	0.630 (2)	0.041 (9)*	
H10G	0.392 (2)	0.449 (5)	0.6864 (19)	0.036 (8)*	
H10E	0.425 (3)	0.802 (6)	0.728 (3)	0.062 (11)*	
H10F	0.436 (2)	0.781 (6)	0.654 (2)	0.053 (10)*	
H10A	0.718 (2)	0.192 (6)	0.760 (2)	0.056 (10)*	
H10I	0.781 (2)	0.915 (5)	0.850 (2)	0.039 (8)*	
H10C	0.568 (3)	0.622 (6)	0.746 (2)	0.055 (11)*	
H10D	0.634 (2)	0.518 (5)	0.7604 (19)	0.034 (8)*	
H10B	0.763 (2)	0.367 (5)	0.7558 (19)	0.033 (8)*	
H10K	0.905 (3)	0.515 (7)	0.816 (3)	0.076 (14)*	
H10L	0.881 (3)	0.528 (6)	0.738 (3)	0.072 (13)*	
H10H	0.389 (3)	0.243 (7)	0.709 (3)	0.077 (13)*	
H10J	0.714 (3)	0.832 (6)	0.788 (2)	0.057 (11)*	
H50A	0.868 (2)	0.928 (5)	0.683 (2)	0.044 (9)*	
H50B	0.803 (3)	1.094 (6)	0.651 (2)	0.063 (11)*	
H50C	0.902 (3)	1.156 (5)	0.701 (2)	0.055 (10)*	

### Atomic displacement parameters (Å^2^)

**Table:**

	*U*^11^	*U*^22^	*U*^33^	*U*^12^	*U*^13^	*U*^23^
O12	0.0148 (7)	0.0379 (8)	0.0204 (7)	0.0037 (6)	0.0089 (6)	0.0030 (6)
O74	0.0245 (8)	0.0298 (7)	0.0219 (7)	−0.0003 (6)	0.0102 (6)	0.0024 (6)
O10	0.0172 (7)	0.0413 (9)	0.0174 (7)	0.0025 (6)	0.0073 (6)	0.0042 (6)
O72	0.0165 (7)	0.0332 (8)	0.0358 (9)	0.0021 (6)	0.0117 (6)	−0.0004 (7)
O102	0.0174 (8)	0.0367 (9)	0.0231 (7)	−0.0010 (7)	0.0065 (6)	−0.0007 (7)
O11	0.0138 (7)	0.0391 (9)	0.0191 (7)	0.0056 (6)	0.0055 (6)	0.0018 (6)
O32	0.0144 (7)	0.0329 (8)	0.0264 (8)	0.0030 (6)	0.0030 (6)	−0.0036 (6)
O34	0.0196 (7)	0.0384 (9)	0.0206 (7)	0.0012 (6)	0.0081 (6)	−0.0031 (6)
O54	0.0235 (8)	0.0373 (8)	0.0233 (8)	0.0024 (7)	0.0049 (7)	−0.0005 (7)
O1	0.0149 (7)	0.0485 (10)	0.0214 (7)	0.0049 (7)	0.0031 (6)	0.0077 (7)
O100	0.0244 (9)	0.0339 (9)	0.0214 (8)	0.0019 (7)	0.0081 (7)	−0.0022 (7)
O105	0.0314 (9)	0.0353 (9)	0.0236 (8)	0.0057 (7)	0.0145 (7)	0.0029 (7)
O52	0.0197 (8)	0.0368 (9)	0.0378 (9)	0.0017 (6)	0.0154 (7)	−0.0023 (7)
O2	0.0150 (7)	0.0467 (10)	0.0282 (8)	0.0038 (7)	0.0075 (7)	0.0045 (7)
O104	0.0268 (8)	0.0426 (10)	0.0290 (8)	0.0020 (7)	0.0159 (7)	0.0003 (8)
O101	0.0279 (9)	0.0394 (10)	0.0288 (9)	−0.0025 (8)	0.0085 (7)	0.0033 (7)
N27	0.0135 (8)	0.0242 (8)	0.0233 (9)	0.0001 (6)	0.0078 (7)	−0.0025 (6)
N41	0.0172 (8)	0.0218 (8)	0.0262 (9)	0.0010 (7)	0.0104 (7)	−0.0017 (7)
N23	0.0164 (8)	0.0250 (8)	0.0203 (8)	0.0000 (7)	0.0074 (7)	−0.0001 (7)
N67	0.0185 (9)	0.0222 (8)	0.0236 (8)	0.0005 (7)	0.0122 (7)	0.0006 (7)
N21	0.0128 (8)	0.0232 (8)	0.0200 (8)	0.0001 (6)	0.0058 (7)	−0.0005 (6)
N61	0.0144 (8)	0.0278 (9)	0.0245 (9)	−0.0001 (7)	0.0094 (7)	0.0000 (7)
N63	0.0190 (9)	0.0281 (9)	0.0224 (8)	−0.0001 (7)	0.0111 (7)	0.0008 (7)
O103	0.0296 (10)	0.0955 (18)	0.0216 (9)	−0.0251 (11)	0.0102 (8)	−0.0029 (10)
N45	0.0164 (8)	0.0253 (9)	0.0234 (9)	0.0010 (7)	0.0086 (7)	−0.0011 (7)
N47	0.0121 (8)	0.0251 (8)	0.0270 (9)	0.0005 (7)	0.0046 (7)	−0.0026 (7)
N43	0.0155 (9)	0.0225 (8)	0.0268 (9)	0.0010 (6)	0.0047 (7)	−0.0012 (7)
N25	0.0146 (8)	0.0250 (8)	0.0187 (8)	0.0013 (6)	0.0038 (7)	0.0010 (6)
N65	0.0147 (8)	0.0231 (8)	0.0210 (8)	0.0011 (6)	0.0073 (7)	0.0015 (7)
C9	0.0166 (9)	0.0211 (9)	0.0177 (9)	−0.0003 (7)	0.0073 (8)	0.0000 (7)
C28	0.0156 (9)	0.0194 (9)	0.0208 (9)	−0.0013 (7)	0.0066 (8)	−0.0014 (7)
C68	0.0194 (10)	0.0170 (9)	0.0242 (10)	−0.0007 (7)	0.0110 (8)	−0.0004 (7)
C64	0.0168 (9)	0.0189 (9)	0.0223 (10)	−0.0015 (7)	0.0090 (8)	−0.0010 (7)
C5	0.0139 (9)	0.0215 (9)	0.0217 (10)	−0.0007 (7)	0.0084 (8)	−0.0001 (7)
C22	0.0156 (10)	0.0245 (10)	0.0221 (10)	−0.0005 (8)	0.0078 (8)	−0.0002 (8)
C4	0.0151 (9)	0.0179 (9)	0.0211 (9)	−0.0003 (7)	0.0066 (8)	−0.0006 (7)
C26	0.0152 (9)	0.0204 (9)	0.0227 (10)	−0.0003 (7)	0.0053 (8)	−0.0022 (8)
C3	0.0161 (9)	0.0222 (9)	0.0214 (10)	0.0000 (7)	0.0056 (8)	0.0008 (8)
C42	0.0134 (10)	0.0236 (10)	0.0307 (11)	0.0011 (7)	0.0076 (8)	−0.0013 (8)
C62	0.0176 (10)	0.0329 (11)	0.0252 (10)	0.0001 (8)	0.0118 (8)	0.0008 (8)
C6	0.0187 (9)	0.0199 (9)	0.0190 (9)	−0.0013 (7)	0.0078 (8)	−0.0009 (7)
C29	0.0128 (9)	0.0200 (9)	0.0206 (9)	−0.0006 (7)	0.0050 (7)	0.0001 (7)
C24	0.0146 (9)	0.0180 (9)	0.0218 (10)	0.0000 (7)	0.0063 (8)	0.0003 (7)
C8	0.0158 (9)	0.0196 (9)	0.0222 (10)	−0.0003 (7)	0.0097 (8)	−0.0005 (7)
C69	0.0151 (9)	0.0193 (9)	0.0225 (10)	0.0004 (7)	0.0086 (8)	0.0003 (7)
C7	0.0145 (9)	0.0196 (9)	0.0193 (9)	0.0016 (7)	0.0042 (7)	−0.0001 (7)
C49	0.0132 (9)	0.0206 (9)	0.0238 (10)	0.0003 (7)	0.0072 (8)	−0.0015 (7)
C48	0.0178 (10)	0.0189 (9)	0.0282 (11)	0.0002 (8)	0.0078 (9)	−0.0021 (8)
C46	0.0167 (10)	0.0204 (9)	0.0291 (11)	−0.0007 (7)	0.0089 (8)	−0.0022 (8)
C30	0.0144 (9)	0.0281 (10)	0.0175 (9)	0.0007 (7)	0.0028 (8)	0.0003 (8)
C44	0.0159 (9)	0.0184 (9)	0.0253 (10)	−0.0011 (7)	0.0078 (8)	−0.0025 (8)
C66	0.0187 (9)	0.0181 (9)	0.0271 (10)	0.0000 (7)	0.0107 (8)	−0.0003 (8)
C71	0.0199 (10)	0.0316 (11)	0.0235 (10)	0.0017 (9)	0.0048 (8)	0.0005 (9)
C31	0.0205 (10)	0.0381 (12)	0.0182 (10)	0.0022 (9)	0.0044 (8)	0.0024 (9)
C51	0.0279 (12)	0.0360 (12)	0.0226 (10)	−0.0008 (9)	0.0119 (9)	−0.0024 (9)
C50	0.0280 (12)	0.0328 (12)	0.0312 (12)	0.0035 (10)	0.0190 (10)	−0.0003 (10)
C33	0.0148 (10)	0.0335 (11)	0.0327 (12)	0.0018 (8)	0.0125 (9)	−0.0035 (9)
C53	0.0146 (10)	0.0324 (11)	0.0376 (13)	0.0031 (8)	0.0023 (9)	−0.0039 (10)
C73	0.0266 (12)	0.0373 (12)	0.0315 (12)	0.0016 (9)	0.0198 (10)	0.0014 (10)
C70	0.0175 (10)	0.0350 (11)	0.0255 (10)	0.0019 (9)	0.0056 (8)	0.0018 (9)

### Geometric parameters (Å, º)

**Table:**

O12—C8	1.366 (2)	N43—C44	1.360 (3)
O12—H12	0.86 (3)	N25—C26	1.370 (3)
O74—C68	1.234 (3)	N25—C24	1.376 (3)
O10—C6	1.359 (2)	N25—C31	1.465 (3)
O10—H10	0.86 (4)	N65—C64	1.365 (3)
O72—C66	1.221 (3)	N65—C66	1.372 (3)
O102—H10A	0.89 (4)	N65—C71	1.465 (3)
O102—H10B	0.85 (4)	C9—C4	1.396 (3)
O11—C7	1.356 (2)	C9—C8	1.383 (3)
O11—H11	0.83 (4)	C9—H9	1.00 (3)
O32—C26	1.229 (3)	C28—C29	1.426 (3)
O34—C28	1.224 (3)	C68—C69	1.415 (3)
O54—C48	1.221 (3)	C64—C69	1.378 (3)
O1—C3	1.333 (3)	C5—C4	1.388 (3)
O1—H1	0.85 (3)	C5—C6	1.386 (3)
O100—H10C	0.83 (4)	C5—H5	0.97 (3)
O100—H10D	0.82 (4)	C22—H22	0.97 (3)
O105—H10E	0.86 (5)	C4—C3	1.481 (3)
O105—H10F	0.88 (4)	C42—H42	0.92 (3)
O52—C46	1.219 (3)	C62—H62	0.96 (3)
O2—C3	1.213 (3)	C6—C7	1.403 (3)
O104—H10G	0.91 (3)	C29—C24	1.364 (3)
O104—H10H	0.93 (5)	C8—C7	1.395 (3)
O101—H10I	0.94 (4)	C49—C48	1.425 (3)
O101—H10J	0.88 (4)	C49—C44	1.370 (3)
N27—C28	1.406 (3)	C30—H30A	0.9800
N27—C26	1.396 (3)	C30—H30B	0.9800
N27—C33	1.481 (3)	C30—H30C	0.9800
N41—C42	1.339 (3)	C71—H71A	0.9800
N41—C49	1.387 (3)	C71—H71B	0.9800
N41—C50	1.458 (3)	C71—H71C	0.9800
N23—C22	1.339 (3)	C31—H31A	0.9800
N23—C24	1.358 (3)	C31—H31B	0.9800
N67—C68	1.403 (3)	C31—H31C	0.9800
N67—C66	1.399 (3)	C51—H51A	0.9800
N67—C73	1.467 (3)	C51—H51B	0.9800
N21—C22	1.342 (3)	C51—H51C	0.9800
N21—C29	1.385 (3)	C50—H50A	0.94 (3)
N21—C30	1.456 (3)	C50—H50B	0.96 (4)
N61—C62	1.336 (3)	C50—H50C	1.00 (4)
N61—C69	1.385 (3)	C33—H33A	0.9800
N61—C70	1.461 (3)	C33—H33B	0.9800
N63—C64	1.353 (3)	C33—H33C	0.9800
N63—C62	1.338 (3)	C53—H53A	0.9800
O103—H10K	0.78 (5)	C53—H53B	0.9800
O103—H10L	0.84 (5)	C53—H53C	0.9800
N45—C46	1.374 (3)	C73—H73A	0.9800
N45—C44	1.375 (3)	C73—H73B	0.9800
N45—C51	1.466 (3)	C73—H73C	0.9800
N47—C48	1.412 (3)	C70—H70A	0.9800
N47—C46	1.396 (3)	C70—H70B	0.9800
N47—C53	1.471 (3)	C70—H70C	0.9800
N43—C42	1.336 (3)		
			
C8—O12—H12	107 (2)	N23—C24—C29	112.27 (18)
C6—O10—H10	108 (3)	C29—C24—N25	122.19 (18)
H10A—O102—H10B	109 (3)	O12—C8—C9	123.47 (18)
C7—O11—H11	111 (2)	O12—C8—C7	116.40 (18)
C3—O1—H1	111 (2)	C9—C8—C7	120.14 (18)
H10C—O100—H10D	97 (3)	N61—C69—C68	132.3 (2)
H10E—O105—H10F	109 (4)	C64—C69—N61	104.82 (17)
H10G—O104—H10H	105 (3)	C64—C69—C68	122.86 (19)
H10I—O101—H10J	99 (3)	O11—C7—C6	121.96 (18)
C28—N27—C33	116.29 (17)	O11—C7—C8	118.61 (18)
C26—N27—C28	126.45 (17)	C8—C7—C6	119.43 (18)
C26—N27—C33	117.24 (17)	N41—C49—C48	131.5 (2)
C42—N41—C49	106.10 (18)	C44—C49—N41	105.08 (18)
C42—N41—C50	126.3 (2)	C44—C49—C48	123.34 (19)
C49—N41—C50	127.5 (2)	O54—C48—N47	121.84 (19)
C22—N23—C24	103.11 (17)	O54—C48—C49	127.2 (2)
C68—N67—C73	117.64 (18)	N47—C48—C49	110.97 (19)
C66—N67—C68	126.75 (17)	O52—C46—N45	121.3 (2)
C66—N67—C73	115.61 (18)	O52—C46—N47	121.2 (2)
C22—N21—C29	106.10 (17)	N45—C46—N47	117.47 (18)
C22—N21—C30	126.55 (18)	N21—C30—H30A	109.5
C29—N21—C30	127.34 (17)	N21—C30—H30B	109.5
C62—N61—C69	106.06 (18)	N21—C30—H30C	109.5
C62—N61—C70	126.25 (19)	H30A—C30—H30B	109.5
C69—N61—C70	127.66 (18)	H30A—C30—H30C	109.5
C62—N63—C64	103.07 (18)	H30B—C30—H30C	109.5
H10K—O103—H10L	110 (5)	N43—C44—N45	126.21 (19)
C46—N45—C44	119.25 (18)	N43—C44—C49	111.87 (18)
C46—N45—C51	119.00 (18)	C49—C44—N45	121.92 (18)
C44—N45—C51	121.74 (18)	O72—C66—N67	121.06 (19)
C48—N47—C53	117.03 (19)	O72—C66—N65	121.9 (2)
C46—N47—C48	127.02 (18)	N65—C66—N67	117.06 (18)
C46—N47—C53	115.92 (18)	N65—C71—H71A	109.5
C42—N43—C44	103.33 (18)	N65—C71—H71B	109.5
C26—N25—C24	119.03 (17)	N65—C71—H71C	109.5
C26—N25—C31	120.56 (18)	H71A—C71—H71B	109.5
C24—N25—C31	120.40 (17)	H71A—C71—H71C	109.5
C64—N65—C66	119.72 (17)	H71B—C71—H71C	109.5
C64—N65—C71	120.76 (17)	N25—C31—H31A	109.5
C66—N65—C71	119.51 (18)	N25—C31—H31B	109.5
C4—C9—H9	122.5 (15)	N25—C31—H31C	109.5
C8—C9—C4	119.88 (18)	H31A—C31—H31B	109.5
C8—C9—H9	117.6 (15)	H31A—C31—H31C	109.5
O34—C28—N27	120.75 (18)	H31B—C31—H31C	109.5
O34—C28—C29	127.67 (19)	N45—C51—H51A	109.5
N27—C28—C29	111.58 (17)	N45—C51—H51B	109.5
O74—C68—N67	121.51 (19)	N45—C51—H51C	109.5
O74—C68—C69	126.7 (2)	H51A—C51—H51B	109.5
N67—C68—C69	111.75 (18)	H51A—C51—H51C	109.5
N63—C64—N65	126.15 (19)	H51B—C51—H51C	109.5
N63—C64—C69	112.08 (18)	N41—C50—H50A	108 (2)
N65—C64—C69	121.77 (18)	N41—C50—H50B	107 (3)
C4—C5—H5	122.4 (16)	N41—C50—H50C	108 (2)
C6—C5—C4	119.37 (19)	H50A—C50—H50B	107 (3)
C6—C5—H5	118.2 (16)	H50A—C50—H50C	115 (3)
N23—C22—N21	113.42 (19)	H50B—C50—H50C	111 (3)
N23—C22—H22	126.9 (17)	N27—C33—H33A	109.5
N21—C22—H22	119.4 (17)	N27—C33—H33B	109.5
C9—C4—C3	121.08 (18)	N27—C33—H33C	109.5
C5—C4—C9	120.66 (18)	H33A—C33—H33B	109.5
C5—C4—C3	118.26 (18)	H33A—C33—H33C	109.5
O32—C26—N27	120.64 (19)	H33B—C33—H33C	109.5
O32—C26—N25	121.57 (19)	N47—C53—H53A	109.5
N25—C26—N27	117.80 (18)	N47—C53—H53B	109.5
O1—C3—C4	112.93 (18)	N47—C53—H53C	109.5
O2—C3—O1	122.85 (19)	H53A—C53—H53B	109.5
O2—C3—C4	124.22 (19)	H53A—C53—H53C	109.5
N41—C42—H42	123 (2)	H53B—C53—H53C	109.5
N43—C42—N41	113.62 (19)	N67—C73—H73A	109.5
N43—C42—H42	123 (2)	N67—C73—H73B	109.5
N61—C62—N63	113.97 (19)	N67—C73—H73C	109.5
N61—C62—H62	122.8 (19)	H73A—C73—H73B	109.5
N63—C62—H62	123.2 (19)	H73A—C73—H73C	109.5
O10—C6—C5	123.93 (18)	H73B—C73—H73C	109.5
O10—C6—C7	115.58 (18)	N61—C70—H70A	109.5
C5—C6—C7	120.49 (19)	N61—C70—H70B	109.5
N21—C29—C28	131.99 (19)	N61—C70—H70C	109.5
C24—C29—N21	105.09 (17)	H70A—C70—H70B	109.5
C24—C29—C28	122.92 (18)	H70A—C70—H70C	109.5
N23—C24—N25	125.54 (18)	H70B—C70—H70C	109.5

### Hydrogen-bond geometry (Å, º)

**Table:**

*D*—H···*A*	*D*—H	H···*A*	*D*···*A*	*D*—H···*A*
O1—H1···O32^i^	0.85 (3)	1.86 (3)	2.672 (2)	161 (3)
O10—H10···O104	0.86 (4)	1.79 (4)	2.643 (2)	174 (4)
O11—H11···O10	0.83 (3)	2.32 (4)	2.709 (2)	109 (3)
O11—H11···O102	0.83 (4)	1.88 (4)	2.680 (2)	161 (3)
O12—H12···O100^i^	0.86 (3)	1.86 (3)	2.702 (2)	166 (3)
O100—H10*C*···O105	0.83 (4)	2.07 (4)	2.851 (3)	157 (4)
O100—H10*D*···O10	0.82 (4)	2.74 (3)	3.286 (2)	126 (3)
O100—H10*D*···O102	0.82 (4)	2.02 (4)	2.798 (3)	158 (3)
O101—H10*I*···N43^ii^	0.94 (4)	1.97 (4)	2.898 (3)	170 (3)
O101—H10*J*···O100	0.88 (4)	2.09 (4)	2.960 (3)	170 (4)
O102—H10*A*···O101^iii^	0.89 (4)	1.87 (4)	2.754 (3)	169 (4)
O102—H10*B*···O103	0.85 (4)	1.85 (4)	2.691 (3)	173 (3)
O103—H10*K*···O34^iv^	0.78 (5)	2.06 (5)	2.832 (3)	168 (5)
O103—H10*L*···N23^v^	0.84 (5)	1.99 (5)	2.823 (3)	168 (4)
O104—H10*G*···O1^vi^	0.91 (3)	2.50 (3)	3.011 (2)	116 (2)
O104—H10*G*···O105	0.91 (3)	2.03 (3)	2.857 (3)	151 (3)
O104—H10*H*···O74^vi^	0.93 (5)	2.00 (5)	2.924 (2)	171 (4)
O105—H10*E*···O74^ii^	0.86 (5)	2.11 (5)	2.952 (2)	169 (4)
O105—H10*F*···N63	0.88 (4)	1.92 (4)	2.798 (2)	173 (4)
C22—H22···O72^vii^	0.97 (3)	2.38 (3)	3.227 (3)	146 (2)
C30—H30*C*···O12^vii^	0.98	2.71	3.247 (3)	115
C31—H31*C*···O2^vi^	0.98	2.40	3.326 (3)	158
C42—H42···O12^viii^	0.92 (3)	2.45 (3)	3.254 (3)	146 (3)
C42—H42···O72	0.92 (3)	2.66 (3)	3.136 (3)	113 (2)
C62—H62···O52^vii^	0.96 (3)	2.24 (3)	3.119 (3)	151 (3)

Symmetry codes: (i) *x*, −*y*+1, *z*−1/2; (ii) *x*, −*y*+2, *z*+1/2; (iii) *x*, *y*−1, *z*; (iv) *x*+1, −*y*+1, *z*+1/2; (v) *x*+1, *y*, *z*; (vi) *x*, −*y*+1, *z*+1/2; (vii) *x*−1, *y*, *z*; (viii) *x*, *y*+1, *z*.
